# How long limbs reduce the energetic burden on the heart of the giraffe

**DOI:** 10.1242/jeb.251092

**Published:** 2025-10-20

**Authors:** Roger S. Seymour, Edward P. Snelling

**Affiliations:** ^1^ School of Biological Sciences, University of Adelaide, Adelaide, SA 5005, Australia; ^2^ Department of Anatomy and Physiology, and Centre for Veterinary Wildlife Research, Faculty of Veterinary Science, University of Pretoria, Onderstepoort, Gauteng 0110, South Africa

**Keywords:** Blood pressure, Cardiac energy, Cardiac output, Efficiency, Evolution, Gravity, Legs, Neck, Scaling

## Abstract

Adult giraffes have high mean systemic arterial blood pressure (MAP) of 200–250 mmHg at heart level, which is more than twice that of most mammals. The high MAP is associated with their long neck, because gravity creates a hydrostatic pressure gradient along the carotid arteries such that every metre of height requires an additional 77 mmHg of blood pressure at heart level, and the head can be over 2 m above the heart. The giraffe's MAP remains high regardless of posture or level of activity, so it creates a significant and unrelenting energy burden on the heart. This study quantifies that burden. Because of high MAP, the energy expenditure of the left ventricle is approximately 16% of the resting whole-body metabolic rate of an adult giraffe, compared with 9% in a normal mammal of the same body mass and a shorter neck. A numerical model is presented that varies the vertical position of the heart in a giraffe's body of fixed height and recalculates the energy used by the left ventricle. If the giraffe had evolved its height by extending the neck alone without elongating the limbs, the estimated cost would be 21%. However, the long limbs, which predate the long neck in giraffe evolution, have raised the level of the heart, thus reducing the required MAP and saving energy. The vertical distance between the heart and the erect head of adult giraffes appears to be the maximum ever evolved among terrestrial vertebrates.

## Teaser

**Summary:** Why do giraffes have such long legs? Because they raise the heart and reduce the high energy cost of pumping blood up the neck against gravity.

## INTRODUCTION

The evolution of the giraffe's long neck permits browsing of foliage that is out of reach of competing species (du Toit, 1990). The exclusive access to the acacia canopy, where crude protein in the young leaves is greater than is available to grazing species, is thought to facilitate breeding throughout the year (Pellew, 1984). Furthermore, the succulent canopy leaves may allow giraffes to be more resistant to drought (Mitchell, 2021). Evolution of sexual dimorphism of the giraffe's neck is purportedly driven by segregation of the sexes in access to high foliage and by fighting among males for access to females (Cavener et al., 2024; Wang et al., 2022).

The giraffe's lifestyle comes with unique energy costs associated with its height. The mass of the neck accounts for 7.0% of body mass in females and 7.9% in males (Mitchell et al., 2013). Because these necks are much heavier than those of grazing artiodactyls of similar body mass, the excess tissue requires more energy to build and maintain. However, a larger direct cost of the neck is the additional energy required by the heart to pump blood to the head against gravity. The left ventricle of the mammalian heart supplies blood to the entire body, while the right ventricle pushes blood through the lungs. The energy that the left ventricle uses increases with body mass primarily because it pumps more blood to meet the demand of a higher whole-body metabolic rate. In addition to this higher systemic cardiac output (CO; l min^−1^), mean systemic arterial blood pressure (MAP; mmHg) at heart level increases significantly in larger and taller mammals (White and Seymour, 2014), apparently related to the increasing vertical distance between the heart and the head (Sandal et al., 2020). The higher CO and greater MAP in larger mammals results in a greater external mechanical work rate by the left ventricle (∝CO×MAP) that must result in a higher myocardial metabolic rate. Kinetic energy is negligible in resting mammals (Blick and Stein, 1977). Finally, the efficiency of the left ventricular myocardium, calculated as the ratio of external work rate and myocardial metabolic rate, decreases in larger mammals (Horrell et al., 2022; Loiselle and Gibbs, 1979; Snelling and Seymour, 2024), which further increases the metabolic demand on the left ventricle. We define the ratio of the metabolic energy expenditure of the left ventricle (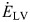) to the resting whole-body energy expenditure (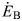) as the ‘relative metabolic cost of the left ventricle’ (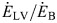), expressed as a percentage (Snelling and Seymour, 2024).

Scaling of cardiovascular and metabolic data from 22 species of mammal shows that 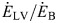 rises significantly with body mass (*M*_B_; kg) according to the equation, 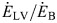
=4.08*M*_B_^0.12^ (Snelling and Seymour, 2024). In percentages, 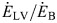 is predicted to increase from about 2.6% in a 20 g mouse to 6.7% in a 60 kg human and to 10.7% in a 3 tonne elephant. The long neck and large vertical distance between the heart and the head in the giraffe necessitates a high MAP that results in an 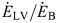 that is greater than predicted for an ordinary mammal of its body size, CO and whole-body metabolic rate (8.9% in a normal 651 kg mammal).

Although the high MAP is necessitated by the length of the neck, it is caused by a high resistance relative to flow rate throughout the entire body. MAP remains high after giraffes lower their heads to the ground to drink (Aalkjær et al., 2025) and it changes little during standing, walking and galloping (Van Citters et al., 1966). Thus, the high energy cost of the heart due to MAP is unrelenting throughout the giraffe's adult life. Although the arteries and veins in the giraffe's neck form an inverted U-shape, the vessels outside the brain case are collapsible and cannot support flow in a siphon. This has been demonstrated theoretically (Pedley et al., 1996), experimentally (Hargens et al., 1987a; van der Walt et al., 2022) and with physical laboratory modelling (Mitchell et al., 2006; Seymour, 2000; Seymour and Johansen, 1987). A strong correlation between MAP at heart level and the vertical distance between the heart and the head among selected mammals, including the giraffe, shows no role of a siphon (Sandal et al., 2020).

The energy and nutrients made available to a giraffe by browsing foliage that is out of reach of other species presumably meets the additional energy costs of having a long neck. However, the neck is not the only adaptation for accessing the canopy. The giraffe's limbs are also long, and the forelimbs are slightly longer than the hindlimbs (van Sittert et al., 2015). Thus, the limbs raise the giraffe's heart, thereby reducing the vertical distance to the head and hence alleviating some of the energetic burden on the heart. Nevertheless, long limbs have disadvantages. A recent analysis of functional morphology concluded that the giraffe suffers a ‘locomotor performance penalty’ because the long lever arms of the forelimbs are not matched by the strength of the shoulder muscles, thereby slowing their maximum speeds and reducing the safety margin for musculoskeletal failure (Basu and Hutchinson, 2022). By comparison, the extinct giraffid *Sivatherium giganteum*, which weighed as much or more than a large male giraffe today but stood only ∼3 m high with a shorter neck and limbs, possessed a stronger relationship between estimated muscle strength and limb length (Basu et al., 2016). Long limbs may well reduce the energy cost of moving a giraffe's body weight a given distance (Pontzer, 2007), but they do not allow giraffes to gather the acceleration and speed needed to outrun a lion (Garland and Janis, 1993; Hirt et al., 2017). The limbs are so long that giraffes are vulnerable during drinking, not only because they lose vigilance when they lower their head to the water but also because they must splay their forelimbs, which makes it cumbersome to rise and run if a predator is sensed. Field observations show that giraffes have the highest levels of vigilance among species at water holes and are most likely to leave a water hole without drinking (Valeix et al., 2008).

The giraffe represents a unique experiment in evolutionary design adaptation. Here, we model the effect of neck and limb lengths on the energetic expenditure of the left ventricle of the heart (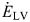). The input variables of the model include the dimensions of the giraffe that affect MAP necessary to perfuse the upright head, the CO that depends on the whole animal's metabolic rate, and the efficiency of the myocardium.

## THE MODEL AND ITS ASSUMPTIONS

### Dimensions of the model giraffe

We calculated the influence of the vertical position of the heart on the relative metabolic cost of the left ventricle in an upright giraffe of fixed height, keeping arterial blood pressure constant at the head. The calculations are scaled to an arbitrary adult body mass of 651 kg based on an earlier cardiovascular review (Seymour and Blaylock, 2000). This body mass is relatively small for adults, which can exceed 1000 kg in females and 1500 kg in males (van Sittert et al., 2015). A 651 kg giraffe is expected to have a standing height of 3.88 m based on the height-to-mass scaling of 17 adult giraffes: *H*=0.429*M*_B_^0.34^, where *H* is height in metres and *M*_B_ is body mass in kg (Østergaard et al., 2013) (equation is derived in Dataset 1, ‘Height and mass’). The level of the aortic semilunar valve in the thorax is marked by the shoulder joint between the scapula and humerus, which is visible from the outside, approximately half-way up the full height of the giraffe (Cavener et al., 2024; Hargens et al., 2007; Silberbauer et al., 2021; Vidal et al., 2020). Thus, heart level is taken as 1.94 m from the ground. To calculate blood pressure at different heights in the arterial system, we add or subtract a value dependent on the densities of blood (1.05 g cm^−3^) and mercury (13.6 g cm^−3^) such that every metre of vertical blood column is equivalent to 77 mmHg gravitational hydrostatic pressure. We ignore the small losses of pressure due to friction as blood passes along the carotid arteries.

### Model values for MAP and CO

The model assumes an arbitrary value of MAP of 214 mmHg, the mean of three studies (Seymour and Blaylock, 2000). This representative value is near others for this animal in the literature (Hargens, 1987; Smerup et al., 2016) and is not controversial. A MAP at heart level of 214 mmHg results in 64 mmHg at the head of our model standing giraffe, a value similar to that of other large mammals (Van Citters et al., 1968; White and Seymour, 2014).

In contrast to MAP, literature values for CO in the giraffe are quite variable. We recently estimated 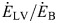 in the giraffe by assuming a heart rate of 102 beats min^−1^ and a CO value of 71.4 l min^−1^; however, we intentionally excluded the giraffe from all regression and statistical analyses of mammals in general (Snelling and Seymour, 2024). We now reject this high value of CO for the giraffe, because we know that the assumed heart mass and stroke volume are too high (Mitchell and Skinner, 2009; Smerup et al., 2016).

Lower values of CO (mean 18.4 l min^−1^, 95% CI 2.5 l min^−1^) were recorded in anaesthetised giraffes, whether recumbent (Goetz et al., 1960; Linton et al., 1999) or with the head held erect with slings (Brøndum et al., 2009; Smerup et al., 2016). The low CO in the most recent study that involved two techniques (Smerup et al., 2016) may be associated with the use of α-chloralose anaesthetic, which, despite assurances of minimal circulatory effects (Covert et al., 1992), can cause progressive depression of heart rate and CO in several other mammal species (Beam et al., 2015; Bollen et al., 1996; Covert et al., 1989; Low et al., 2016). A common effect of α-chloralose is blood acidosis, indicating reduced gas exchange either by reduced CO or by respiratory depression, or both. Reduced CO and acidosis were noted in giraffes anaesthetised with α-chloralose (Smerup et al., 2016) or halothane (Linton et al., 1999). We suspect that CO measurements in anaesthetised giraffes are unnaturally low due mainly to depressed heart rate. Telemetered cardiovascular variables in unrestrained and conscious mammals are considered to represent the natural condition (Poulsen et al., 2018). Heart rates evident in traces measured by telemetry from conscious, unrestrained standing or walking giraffes in the savanna (Van Citters et al., 1966) are 2–3 times higher than in anaesthetised giraffes (Smerup et al., 2016). Heart rates ‘…around 40 beats min^−1^ in undisturbed quietly standing giraffes…’ proposed by Smerup et al. (2016) to be documented in a publication by the Van Citters group (Van Citters et al., 1969) do not appear there. Rather, all data records in that publication refer to measurements of recumbent giraffes during instrument implantation or in the minutes after standing when heart rates were generally above 100 beats min^−1^. There are no heart rates around 40 beats min^−1^ in conscious standing giraffes in any other Van Citters et al. publications (Van Citters et al., 1966, 1968).

It has been postulated that a low CO might be compensated for by a high oxygen extraction, i.e. a greater difference in oxygen content between arterial and mixed venous blood (Smerup et al., 2016). This seems unlikely because it is contrary to a general decrease in oxygen extraction in larger resting mammals (Calder, 1996) and a large oxygen extraction at rest would compromise greater extraction that normally occurs during aerobic exercise (Joyner and Casey, 2015). Nevertheless, the question is unresolved because there are no available measurements of oxygen extraction in giraffes.

Low experimental CO values are also inconsistent with expected and measured resting metabolic rates (RMR) in giraffes. The scaling equations for CO and RMR can predict each other. Allometry among mammals reveals that CO=0.187*M*_B_^0.81^ (Stahl, 1967) and RMR=8.07*M*_B_^0.69^ (White and Seymour, 2003), therefore CO would be 35.6 l min^−1^ and RMR would be 705 ml O_2_ min^−1^ in a 651 kg animal. However, giraffes are artiodactyls, which have higher RMR than other mammals because their gut fermentation prevents them from becoming post-absorptive (Hayssen and Lacy, 1985). Scaling of RMR for seven species of artiodactyls (see Dataset 1, ‘Metabolic rates’) predicts that a 651 kg giraffe would consume 1683 ml O_2_ min^−1^ (White and Seymour, 2003), similar to the actual mean of 1557 ml O_2_ min^−1^ measured in three giraffes (average *M*_B_=597 kg) under resting conditions (Langman et al., 1982). The ratio of RMR in giraffes over mammals in general is 2.39 (=1683/705). If CO is directly proportional to RMR, then CO would be 85.0 l min^−1^ (=2.39×35.6), a very high value, not a low one.

We now recommend an intermediate mean CO measured from conscious, standing giraffes, 41.8 l min^−1^ (Goetz et al., 1960) in our present model, because it comes closer to expectations based on RMR and is not affected by anaesthesia. However, we also report results for both high and low values of CO. Importantly, the level of the heart in the model affects only the MAP required to perfuse the head; the relative changes in heart position are independent of CO.

### Calculation of energy expenditure of the left ventricle

**Fig. 1.**
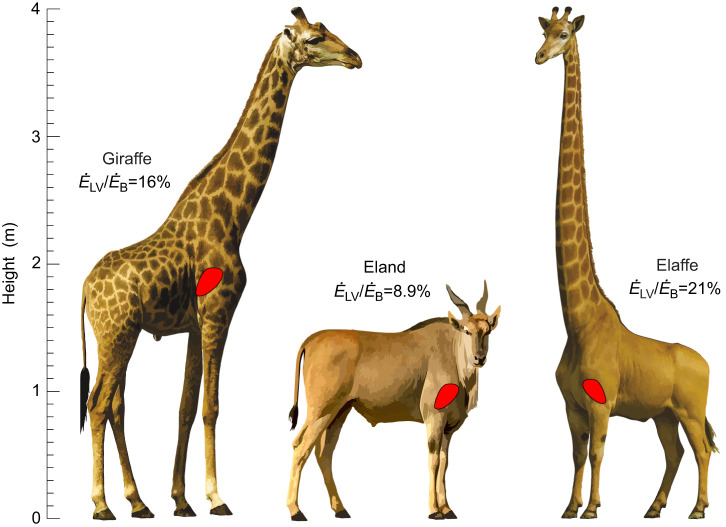
**Scaled illustrations of giraffe and eland, showing the approximate location of the hearts.** The ‘elaffe’ is a chimera that reaches the same height as the giraffe, but by elongation of the neck only. Giraffe and eland heart dimensions are based on an allometric equation (Mitchell and Skinner, 2009). The metabolic energy expenditure of the left ventricle in relation to whole-body metabolic rate (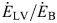) of the giraffe and elaffe is calculated from the present model and that of the eland is derived from allometry of mammals in general (Snelling and Seymour, 2024). All calculations are standardised to a body mass of 651 kg and a resting metabolic rate (RMR) of 1683 ml O_2_ min^−1^.

We convert MAP into pressure energy content of the arterial blood (J l^−1^), using unit conversions of 1 mmHg=0.133 kPa=0.133 J l^−1^. Therefore, 214 mmHg is equivalent to 28.5 J l^−1^. CO is taken as 41.8 l min^−1^ which equals 0.70 l s^−1^. The external work rate of the left ventricle for our resting giraffe is thus 28.5 J l^−1^×0.70 l s^−1^=19.9 J s^−1^=19.9 W. The metabolic energy used by the left ventricle is much more than the external work rate because cardiac muscle is much less than 100% efficient at converting chemical energy into useful external mechanical energy transferred to the blood exiting the left ventricle (Sörensen et al., 2020). Applying the empirical equation for efficiency (η; %) of the mammalian heart, η=32*M*_B_^−0.06^ (Horrell et al., 2022; Snelling and Seymour, 2024), a typical 651 kg mammal has a heart that is 22% efficient, and the metabolic energy expenditure of the left ventricle (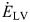) is therefore 90.5 W. (Incidentally, this is more than the whole-body metabolic rate of a resting adult human, ca. 80 W; Snelling and Seymour, 2024.) The resting whole-body energy expenditure (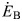) of the giraffe can be estimated from the resting O_2_ consumption rate (RMR), where 1 ml O_2_ (STP) min^−1^=0.348 W (assuming an intermediate respiratory quotient of 0.88). A 651 kg giraffe consumes 1683 ml O_2_ min^−1^, based on an allometric equation (RMR=12.41*M*_B_^0.76^) derived from seven species of resting artiodactyls (all ruminants) (White and Seymour, 2003) (see Dataset 1, ‘Metabolic rates’). Converted to energy units, giraffe 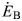
=586 W. Therefore, the relative energy cost of our giraffe's left ventricle (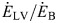) is approximately 16% of its resting whole-body metabolic rate (Fig. 1), a very high value compared with the 8.9% calculated above for a normal 651 kg mammal (Snelling and Seymour, 2024). If giraffes had lower resting metabolic rates comparable to those of non-ruminant mammals (White and Seymour, 2003), 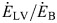 would be well above 30%.

### Effect of heart elevation on energy expenditure

**Fig. 2.**
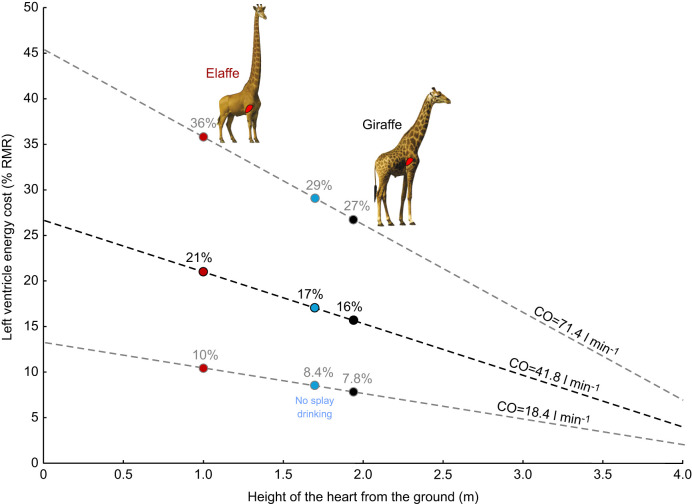
**Results of models showing the relative metabolic cost of the left ventricle (**
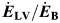
**) in relation to the elevation of the heart above the ground.** All three models assume a fixed standing height of 3.88 m, a body mass of 651 kg and a whole-body RMR of 1683 ml O_2_ min^−1^. The heart in the model giraffe is 1.94 m above the ground and the mean systemic arterial blood pressure (MAP) is 214 mmHg. The three lines show the effects of changing the elevation of the heart and changing assumed cardiac output (CO) between 18.4, 41.8 and 71.4 l min^−1^, based on the range of literature values (see ‘Model values for MAP and CO’ for details). With the intermediate CO, 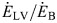 in the normal giraffe is 16% (black dot). However, if the forelimbs of the giraffe were the same length as those of an eland and the neck correspondingly longer, 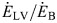 would rise to 21% (red dot). If the limbs were shorter to avoid splaying during drinking (see Fig. 3), 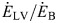 would be 17% (blue dot).

The numerical model permits recalculation of 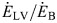 if the heart were repositioned vertically in a giraffe's body of fixed height (see Dataset 1, ‘Model’). If the heart were higher in the body, the MAP required would be less, but the limbs would be longer. Conversely, if the heart were lower in the body, the neck would be longer, and the cost of the heart would be higher. To compare the giraffe with a mammal of more typical proportions, we chose a nominal 651 kg African common eland, *Taurotragus oryx*, standing 1.53 m at shoulder height (Kingdon, 2018) and with a heart approximately 1 m above the ground (Fig. 1). Further, we created a chimera (called the ‘elaffe’) with the body and legs of the eland and the neck of a giraffe reaching to the same height. According to scaling of 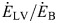 in normal mammals (Snelling and Seymour, 2024), the left ventricle of a 651 kg eland would cost 8.9% of its RMR. According to our model, the standard giraffe's heart, producing a MAP of 214 mmHg, would cost 16%, and the long-necked elaffe, producing 286 mmHg, would cost 21%. The relationship between 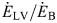 and the elevation of the heart above the ground is a straight line, the slope of which depends on CO (Fig. 2).

**Fig. 3.**
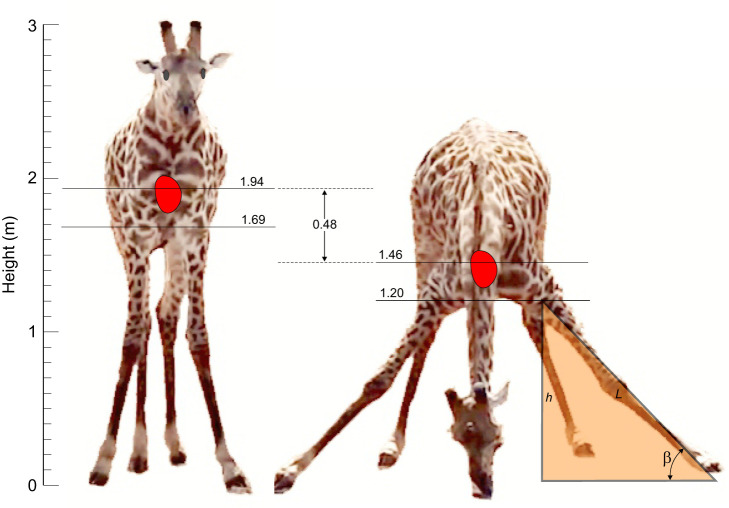
**Scaled illustrations based on video footage of a giraffe standing and drinking at a water hole (**www.youtube.com/watch?v=JMk9dPzEvxA). The heart is located at the level of the shoulder joint, and the height of the elbow joint (*h*) is determined from a triangle incorporating the length of the forelimb axis (*L*) and the angle of the limb to the ground (β). The height scale is determined by the elevation of the heart in our model 651 kg giraffe, assuming proportionality with the photographed animal (van Sittert et al., 2015).

We could ask what 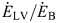 would be if the forelimbs were just short enough to eliminate the need to splay them during drinking but with a longer neck to achieve the same height as the ordinary giraffe. The level of the elbow between the humerus and radius marks the bottom of the thorax, which is the landmark we used for measuring the effects of splaying the forelimbs during drinking. The heights of the elbow and shoulder joints were first calculated from forelimb length allometry for a standing giraffe, which showed constant proportions of the humerus, radius and metacarpus (cannon bone) through postnatal ontogeny (van Sittert et al., 2015). Missing lengths for the carpels (wrist) and phalanges (fingers) were estimated from photographs of mounted skeletons in relation to the photographed radius length. To obtain forelimb height, all bones were considered vertical, except for the humerus, which is oriented diagonally between the shoulder and elbow and therefore its height was estimated as one-half of bone length. The proportions of the bones to the total height of the forelimb to the shoulder are the diagonal humerus 13.0%, vertical radius 38.6%, vertical carpels 3.4%, vertical metacarpus 35.9% and nearly vertical phalanges 9.1%. The ratio of elbow height to shoulder height was 0.87. Thus, for our 651 kg giraffe with a shoulder height of 1.94 m, elbow height was 1.69 m, or 0.25 m below heart level (Fig. 3).

The effect of drinking with splayed forelimbs on heart position was determined from nine public domain photographs taken directly in front of giraffes while they were drinking at ground level with their legs splayed to the side (Fig. 3). The telephotographs minimised parallax, so we assumed two-dimensionality. Relative distances within each image were measured with ImageJ software in raw pixel numbers because the photographs lacked scales. The images were then standardised to our model by assuming that the forelimb length (*L*), from hoof to elbow is 1.69 m in the standing giraffe (Fig. 3). The angle (β) between the forelimb axis (hoof to elbow) and a line between the two hooves allowed calculation of the elbow height above the ground (*h*), relative to the length of the forelimb axis (*L*): *h*=*L*×sinβ (see Dataset 1, ‘Drinking posture’). The length *L* visible in the drinking giraffe is somewhat shorter than the maximum forelimb length, because the wrist can be bent slightly during splaying and the radius appears shorter. This was evident in the ratio of forelimb distances (elbow–wrist/wrist–hoof) decreasing at greater values of β. The value of β from the right and left limbs of each giraffe was averaged [mean 45.7 deg (0.80 rad), 95% CI 2.2 deg, *n*=9] and elbow height calculated (mean 1.20 m, 95% CI 0.04 m, *n*=9). The difference in elbow height between standing and splaying was 0.48 m, and the heart would be lowered by the same distance. However, for the giraffe to drink without forelimb splaying, the heart would not have to be lowered by 0.48 m, but only by one-half of that distance (0.24 m), assuming the neck were increased by the same vertical distance as the forelimbs shortened. The overall effect would be a modest +18 mmHg increase in MAP to 232 mmHg and 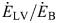 would increase slightly to 17% (cf. 16% in the model giraffe; Fig. 2).

## DISCUSSION

### Evolution of heart position

This study evaluates the cardiac energy burden that the giraffe must endure to reach high vegetation. It also assesses how that burden has been partly alleviated by the co-evolution of long limbs. We have estimated that the giraffe's left ventricle uses 16% of its RMR, compared with 8.9% in a mammal of the same body mass but without a long neck (Fig. 1). The extra energy cost of having the long neck is presumably offset by the energy, nutrients and water gained by having nearly exclusive access to the acacia canopy. If the giraffe had reached the same height by lengthening the neck only, the cost would have risen to 21%. This demonstrates the energetic value of long limbs in raising the heart and reducing the cardiac work necessary to perfuse the brain. Lengthening of the limbs preceded that of the neck during evolution of the Giraffidae, as evident from increasing ratios of forelimb length to neck length in fossil giraffids, from 1.43 in *Canthumeryx* (16 mya), to 1.70 in *Palaeotragus* (11 mya) and 2.15 in *Samotherium* (7 mya) (see fig. 18.5 in Mitchell, 2021). It is possible that this pattern of evolution was influenced by the energy cost of the circulation, but it could also be a coincidence. Today, the ratio of limb/neck length is 0.96 in *Giraffa*, showing nearly equal contributions to achieving height. The forelimbs of extant giraffes are still too long to permit drinking without splaying or flexing the wrist. However, our analysis indicates that the ability to drink without splaying could have occurred with a small additional cardiac cost (Fig. 2).

### Limitations on heart position

The present analysis might make us question why the giraffe's heart did not evolve out of the thorax to reside high in the neck, for this position would greatly reduce its work against gravity. Aside from the obvious skeletal elements in the way, an important limitation is that the heart must be on the same level as the lungs and deliver blood to them at a low pressure. Air pressure in the alveoli of the lungs is always near atmospheric (0 mmHg compared with blood pressure), so if pulmonary blood pressure is too high, it can force fluid through the thin capillary and alveolar walls into the gas spaces (cardiogenic pulmonary oedema), which can impede oxygen uptake into the blood (Burggren, 1982). Normal pulmonary blood pressure is below about 20 mmHg in air-breathing vertebrates for this reason (Johansen, 1972; Snelling et al., 2015). If it rises above 25 mmHg in humans, serious pulmonary oedema is likely (Pannu et al., 2024). The critical pulmonary capillary pressure (beyond which alveolar fluid would accumulate) is 27 mmHg in the giraffe (Hargens et al., 1987b). This limit would be reached with a heart only 35 cm above its present position, because gravity alone would create a hydrostatic pressure of 27 mmHg at the bottom of both the pulmonary arteries and veins.

### Limitations on vertical neck length in terrestrial vertebrates

Discussion of the rising energy cost in longer-necked terrestrial vertebrates would not be complete without mentioning sauropod dinosaurs, some of which had exceptionally long necks. Compared with the 2.4 m neck of the largest giraffes, the longest sauropod neck is estimated to have been 15 m in *Supersaurus* (Taylor and Wedel, 2013). If such a neck were held erect, the hydrostatic component of MAP would have been 15×77=1155 mmHg. Assuming the heart were positioned about 2 m below the base of the neck and a perfusion pressure must be added to achieve blood flow to the brain, MAP would have been about 1350 mmHg. The heart of a shorter sauropod requiring a MAP of 750 mmHg was estimated to consume energy at a rate approximately equal to the entire rest of the body (Seymour, 2009), but it may have been even higher because of reduced efficiency in thicker-walled hearts (Horrell et al., 2022; Snelling and Seymour, 2024). The energy problem of high MAP in erect sauropod necks was pointed out 50 years ago (Seymour, 1976), but potential solutions to the problem, including accessory hearts, the siphon principle, temporary stasis of cranial blood flow or maintaining blood flow in a ‘G-suit’ skin have always failed scrutiny (Seymour, 2016). It is telling that the hydrostatic pressure at the base of the neck of the largest giraffe has never been exceeded by the largest theropod and ornithopod dinosaurs, or in the largest extinct mammals, *Mammuthus* and *Paraceratherium*, even when rearing (Seymour, 2016). We therefore conclude that the largest sauropods could not raise their necks much higher than their shoulders where the required MAP to perfuse the head would be similar to that of large giraffes.

### Declaration of AI use

We did not use AI-assisted technologies in creating this article other than creating the illustration of the ‘elaffe’.

## Supplementary Material

10.1242/jexbio.251092_sup1 Supplementary information

Dataset 1. Data and models
