# Supplementary material for: How long limbs reduce the energetic burden on the heart of the giraffe
Source: J Exp Biol. 2025 Oct 20;228(20):jeb251092. doi: 10.1242/jeb.251092 (PMC12582410; doi:10.1242/jeb.251092)
Supplement: Supplementary information [file jexbio-228-251092-s1.pdf]

Dataset 1. Data and models

Available for download at
https://journals.biologists.com/jeb/article-lookup/doi/10.1242/jeb.251092#supplementary-data

Journal of Experimental Biology • Supplementary information
